# Development of Body Dissatisfaction in Women and Men at Different Educational Levels During the Life Course

**DOI:** 10.1007/s12529-023-10213-x

**Published:** 2023-08-17

**Authors:** Elena Rosenqvist, Hanna Konttinen, Noora Berg, Olli Kiviruusu

**Affiliations:** 1 Faculty of Social Sciences, University of Helsinki, P.O. Box 42, 00014 Helsinki, Finland; 2 Department of Public Health and Welfare, Finnish Institute for Health and Welfare, P.O. Box 30, 00271 Helsinki, Finland

**Keywords:** Body image development, Gender, Socioeconomic position, Cohort study

## Abstract

**Background:**

Our study examines the rarely investigated associations between body dissatisfaction and educational level over the life course in women and men.

**Methods:**

A Finnish cohort (*N* = 1955) was followed by questionnaires at ages 22, 32, 42, and 52. Body dissatisfaction was measured by asking the respondents to evaluate their appearance using five response options. Analyses were done using logistic regression, while latent class analyses were used to identify classes of body dissatisfaction trajectories over the life course.

**Results:**

Body dissatisfaction increased with age in women and men. Among men, body dissatisfaction was related to lower education at the ages of 32 and 42. Also, men with lower education were more likely to maintain a less positive body image over the life course. In women, increasing body dissatisfaction during the life course was associated with lower education.

**Conclusions:**

Differences in body dissatisfaction based on educational level are important to take into account in public health actions aiming to reduce socioeconomic inequalities in health and well-being.

## Introduction

In modern Western society, people live surrounded by strict appearance norms [1, 2], high stigmatization of obesity [3], and daily exposure to a media imagery full of flawless-looking women and men [4]. This cultural environment, with unrealistic appearance expectations, can easily expose an individual to problems in body image (i.e., an individual’s perceptions, thoughts, and feelings about his or her own body) [4, 5]. The research of body image has traditionally concerned young women, but during the last decades, the research field has increasingly shifted towards studying the body image of both men and women at different ages [6, 7]. Although appearance standards as well as the concern about one’s body and looks have been proposed to be more upper-class phenomena [8], the socioeconomic patterning of body dissatisfaction has remained relatively rarely investigated [7, 9].

A wide range of studies considering women’s body image demonstrate that women experience a high amount of body dissatisfaction and that the tendency remains remarkably constant through the life span [10–14]. Women generally report experiencing more body dissatisfaction than men throughout life course, although the importance women attach to their physical appearance declines with age [11, 14, 15]. A central aspect in women’s body dissatisfaction is a regular desire to be leaner [7, 10], which reflects the Western body ideal associating slimness with social attractiveness and youth [1]. Accordingly, higher body mass index (BMI) has been found to have a stronger negative effect on women’s body image [16] and self-esteem [17] over the life course compared to men.

The study regarding men’s body image is relatively new but has been the subject of growing interest in the twenty-first century [6, 7], reflecting the societal change, in which men are under increased social pressure to look muscular and toned [2]. Body dissatisfaction seems to appear in a more complex way in men than in women, as men’s body concerns seem to change qualitatively with aging: generally, young men desire to be more muscular, whereas later in adulthood, men desire to be slimmer [15, 18, 19].

Despite the significant expansions in body image research, research on the associations between body dissatisfaction and socioeconomic status (SES) is limited, and in most studies regarding the level of body dissatisfaction, SES gradients have been excluded from the analyses [7, 9]. Given that body concerns and appearance standards have been shown to be SES-related, it is reasonable to assume that also body dissatisfaction would have associations with SES. In those studies taking SES into account, the results have varied, but most of them indicate an association between higher body dissatisfaction and higher SES. Education is one of the commonly used measures of SES [20, 21]. Millstein et al. [22] found weight dissatisfaction to be associated with higher education in an adult population of women and men of wide age range. In a couple of studies, body dissatisfaction has been related to higher education among middle-aged women [23, 24]. In some studies, the association between body dissatisfaction and higher SES has been found among men exclusively: Matthiasdottir et al. [25] found body dissatisfaction to be associated with higher education in men but not in women in an Icelandic population. In the study of von Lengerke and Mielck [26], body dissatisfaction was related to higher SES, but only among overweight and obese men. Women and men in higher SES groups have also been shown to be more likely to perceive themselves as overweight [8]. The association of body concerns with upper social classes has been explained by using Pierre Bourdieu’s theory of social distinction, assuming that in upper social classes, thinness can be used as a marker of social distinction, making the body a project to achieve higher status [1, 27]. However, there are also a few contradictory findings indicating that in adolescence and young adulthood, body dissatisfaction would be associated with lower parental SES among females [28, 29] but not in males [28].

In our study, the aim is to examine the associations between body dissatisfaction and SES (measured through education) among women and men in a 30-year follow-up. Body image in this study is measured by an assessment of satisfaction with appearance, which is one element with which the broad concept of body image has been conceptualized and operationalized in the research literature [7]. Also, we examine the role of BMI in explaining the associations between body image and SES. Our three research questions are:

1. Is body dissatisfaction related to education in women and men at different ages?
2. Are the longitudinal trajectory groups of body dissatisfaction related to education in women and men?
3. Does BMI explain the associations between body dissatisfaction and education in women and men?

Given that body dissatisfaction has traditionally been investigated among young women, our study is in line with the current trend of extending the research to men and women at different ages. However, it is essential to note that the previous research results of age-varying changes in body image, in both women and men, are generally based on cross-sectional studies comparing different age groups, instead of following the same cohort by age [7, 16]. Aging causes changes in the body that can affect body image and that cannot be fully captured in cross-sectional studies comparing different age groups. There has been a call for longitudinal cohort studies of body image, to better control the effect of historical changes in body ideals [7]. The prospective study design of our study enables us to compare the development of body image between women and men and its associations with education during the life span. Based on the previous literature, we hypothesize that body dissatisfaction would be related to higher education. Due to the limited amount of previous literature, specific hypotheses taking gender and age into account are not made.

## Methods

### Subjects

The study is a part of the Stress, Development and Mental Health (TAM) project [30]. The study population (practically all Caucasians) consisted of all Finnish-speaking ninth-grade pupils (*N* = 2269) attending comprehensive school in 1983 in Tampere, an industrial and university city in Southern Finland, with some 166 000 inhabitants at that time. In 1983, 2194 pupils (response rate: 96.7%), with a mean age 15.9 years (SD 0.3 years), completed a questionnaire at school. Participants of the 1983 baseline (*N* = 2194) have been followed up by postal questionnaires at ages 22 (*N* = 1656, 75.5%), 32 (*N* = 1471, 67.0%), 42 (*N* = 1334, 60.8%), and 52 (*N* = 1160, 52.9%). The question measuring body dissatisfaction was included in the survey for the first time in 1989, due to which in the present study, we used data of only those participants who took part at least one of the follow-ups between ages 22 and 52 (*N* = 1955).

### Measures

#### Body Dissatisfaction

The question measuring body dissatisfaction in the questionnaire (*How do you feel about your appearance?*) was a part of depression measure R-BDI [31], a Finnish modification of the 13-item Beck Depression Inventory [32], in which each item is supplemented with a response option indicating positive mood. The question regarding body dissatisfaction had five response options (*I am fairly satisfied with my appearance/There are no physical traits that bother me in my appearance/I am worried that I look unattractive/I feel that I look ugly/I am sure that I look ugly and disgusting*). Due to the small number of participants in the two upper categories, a dichotomous body dissatisfaction variable was constructed. The first two answer options were combined into the category of not having reported body dissatisfaction (no) and the last three options into the category of having reported body dissatisfaction (yes). In addition, a four-category variable was constructed for the longitudinal latent class analyses, with the first three categories as in the original variable (1, *I am fairly satisfied with my appearance;* 2, *There are no physical traits that bother me in my appearance;* 3, *I am worried that I look unattractive) and the last two answer options combined into one category;* 4, *I feel that I look ugly/I am sure that I look ugly and disgusting*).

#### Socioeconomic Status (SES)

SES was measured by self-reported education, which is one of the commonly used SES factors. Education as a SES indicator has been shown to reflect differences in awareness, the ability to turn information into behavior and to take control over one’s life [21], which is why we considered education a suitable SES indicator for considering issues related to body image. For 22-year-olds, educational level was measured as a dichotomous variable (graduated/not graduated from high school). At ages of 32–52, educational level was measured on an eight-point scale from “no vocational education” to “post-graduate university degree,” and the answer options were grouped into those who had completed “vocational education or less,” those who had “lower college” degree, and those who had “upper college” degree. For the latent class analyses, an aggregate education variable was constructed, measuring whether the respondent had completed at least lower college education by the age of 52 (yes/no).

#### Control Variables

In the analyses, we controlled for BMI [self-reported weight (kg) by the square of self-reported height (m); coded as missing for women who reported being pregnant (*n* = 34) at the time of the follow-up], marital status (married or cohabiting/neither married nor cohabiting), and having children (yes/no). Aggregate control variables were constructed for longitudinal analyses. The aggregate variable for BMI indicated the mean BMI for a participant from the follow-ups at the ages of 22, 32, 42, and 52. The aggregate variable for marital status indicated whether or not a participated had been “married or cohabiting” in most of the follow-ups between the ages of 22 and 52 (yes/no). The aggregate variable for having children indicated whether a participant had reported having children in any of the follow-ups (yes/no).

### Statistical Analyses

Data were analyzed using SPSS version 28.0, except for latent class analyses that were run with Mplus 8.7 [33]. The distributions of the categorical variables were presented as percentages, and for the continuous variables, the means and standard deviations were calculated. The main analyses were performed separately for women and men. Gender difference in body dissatisfaction across all timepoints as well as the effect of time on body dissatisfaction was analyzed using random effects models.

The associations between dichotomous body dissatisfaction variables and education were visualized using bar charts and tested using binary logistic regression analyses. Three different logistic regression models were done for women and men at every age phase. In model 1, the association of body dissatisfaction with education was examined without any adjustments. In model 2, BMI was added as a predictor to the model, and in model 3, marital status and having children were further added as control variables. For odds ratios (OR), 95% confidence intervals (CI) were calculated. Interaction terms (gender × education) were entered to the models to examine gender differences in the effects. In addition, mixed effects models were used to analyze whether there were significant changes in the association between education and body dissatisfaction between timepoints — these analyses were only performed using follow-ups from age 32 onwards, since the measure of education was different in the first follow-up at 22 years.

In the next step of the analyses, participants were grouped according to their longitudinal life-course trajectories of body dissatisfaction using longitudinal latent class analysis (LLCA). The LLCAs were based on the four-category variable of body dissatisfaction. To determine the best solution (number of classes), different statistical criteria were used (see Appendix Table 4), while emphasis was also placed on relevant and informative interpretation of the classes. Only cases for whom there were at least two (out of four) non-missing values for body dissatisfaction variables were included in the LLCA, leaving 1619 cases in these analyses. After the best solution was established, cases were assigned to the latent classes according to their most likely class memberships.

**Table 1:** Distributions of study variables

	Women (*N* = 1004)		Men (*N* = 951)	
Variable	*N*	Mean (SD)/*N* (%)	N	Mean (SD)/*N* (%)
Body dissatisfaction, 22 y (yes)^a^	885	126 (14.2%)	761	57 (7.5%)
Body dissatisfaction, 32 y (yes)^a^	804	142 (17.7%)	664	50 (7.5%)
Body dissatisfaction, 42 y (yes)^a^	731	146 (20.0%)	599	50 (8.3%)
Body dissatisfaction, 52 y (yes)^a^	646	145 (22.4%)	511	61 (11.9%)
Education, 22 y	890		766	
Not graduated high school		407 (45.7%)		432 (56.4%)
Graduated high school		483 (54.3%)		334 (43.6%)
Education, 32 y	798		665	
Vocational education or less		310 (38.8%)		358 (53.8%)
Lower college		341 (42.7%)		178 (26.8%)
Upper college		147 (18.4%)		129 (19.4%)
Education, 42 y	728		596	
Vocational education or less		235 (32.3%)		277 (46.5%)
Lower college		326 (44.8%)		185 (31.0%)
Upper college		167 (22.9%)		134 (22.5%)
Education, 52 y	645		508	
Vocational education or less		194 (30.1%)		208 (40.9%)
Lower college		299 (46.4%)		177 (34.8%)
Upper college		152 (23.6%)		123 (24.2%)
At least lower college education completed 22–52 y	923	638 (69.1%)	837	435 (52.0%)
*Control variables*				
BMI, 22 y	874	21.60 (3.03)	759	23.02 (2.64)
BMI, 32 y	758	23.76 (4.31)	662	25.27 (3.61)
BMI, 42 y	716	25.53 (5.03)	596	26.81 (4.39)
BMI, 52 y	640	27.25 (5.52)	510	28.16 (4.75)
Mean BMI 22–52 y	1955	24.26 (4.03)	1955	25.52 (3.53)
Married or cohabiting, 22 y	890	344 (38.7%)	766	154 (20.1%)
Married or cohabiting, 32 y	803	619 (77.1%)	664	484 (72.9%)
Married or cohabiting, 42 y	734	550 (74.9%)	600	481 (80.2%)
Married or cohabiting, 52 y	648	480 (74.1%)	512	400 (78.1%)
Married or cohabiting in most of the follow-ups	1955	765 (76.2%)	1955	671 (70.6%)
Having children, 22 y	890	81 (9.1%)	765	31 (4.1%)
Having children, 32 y	795	493 (62.0%)	654	350 (53.5%)
Having children, 42 y	734	568 (77.3%)	600	446 (74.3%)
Having children, 52 y	634	513 (80.9%)	504	404 (80.2%)
Having children in any of the follow-ups	1955	713 (88.8%)	1955	617 (91.0%)

^a^The dichotomous body dissatisfaction variable was constructed for each age stage as recoding the answer options “I am fairly satisfied with my appearance” and “There are no physical traits that bother me in my appearance” as not having body dissatisfaction and the answer options “I am worried that I look unattractive”, “I feel that I look ugly” and “I am sure that I look ugly and disgusting” as having body dissatisfaction

In the final phase of the analyses, the longitudinal associations between body dissatisfaction and education were examined using multinomial logistic regression analyses. Membership of longitudinal latent classes of body dissatisfaction was predicted by the aggregate education variable that indicated whether the respondent had completed at least lower college education by the age of 52. Again, three different models were run for women and men separately. In model 1, the unadjusted association between body dissatisfaction classes and the aggregate education variable was examined. Model 2 was adjusted for mean BMI across time points, and model 3 was further adjusted for the aggregate variables of marital status and having children. For relative risk ratios (RRR), 95% confidence intervals (CI) were calculated. To examine gender differences in the associations, interaction terms (gender × aggregate education) were added to the models.

## Results

Descriptive statistics are given in Table 1. Body dissatisfaction was more common in women than in men throughout the follow-up, and the odds ratio of the female sex on body dissatisfaction across timepoints was significant (OR = 2.35, *p* < 0.001). Lower college education was the most common educational group for women at the ages of 32–52, and vocational education or less was the most common educational group for men at the ages of 32–52 (Table 1). BMI increased with age in women and men.

**Table 2:** Body dissatisfaction at ages 22, 32, 42, and 52 by educational level among women and men. Logistic regression

	**Women**			**Men**		
	**Model 1**	**Model 2**	**Model 3**	**Model 1**	**Model 2**	**Model 3**
	**OR (CI 95%)**	**OR (CI 95%)**	**OR (CI 95%)**	**OR (CI 95%)**	**OR (CI 95%)**	**OR (CI 95%)**
22 y						
Not graduated high school						
Graduated high school	0.80 (0.55–1.18)	0.93 (0.63–1.38)	1.05 (0.69–1.61)	0.93 (0.54–1.61)	0.99 (0.57–1.72)	0.95 (0.55–1.66)
32 y						
Vocational education or less						
Lower college	1.07 (0.71–1.63)	1.30 (0.83–2.03)	1.32 (0.84–2.08)	0.75 (0.38–1.45)	0.74 (0.38–1.46)	0.76 (0.38–1.53)
Upper college	0.99 (0.58–1.70)^a^	1.38 (0.78–2.45)^a^	1.44 (0.80–2.59)^a^	0.23* (0.07–0.75)^a^	0.26* (0.08–0.87)^a^	0.26* (0.08–0.87)^a^
42 y						
Vocational education or less						
Lower college	0.90 (0.59–1.37)	0.87 (0.55–1.37)	0.89 (0.56–1.40)	0.74 (0.39–1.39)	0.79 (0.41–1.52)	0.83 (0.43–1.61)
Upper college	0.88 (0.53–1.45)^a^	1.07 (0.62–1.83)^a^	1.10 (0.63–1.90)^a^	0.18** (0.05–0.60)^a^	0.23* (0.07–0.78)^a^	0.25* (0.07–0.86)^a^
52 y						
Vocational education or less						
Lower college	1.11 (0.72–1.72)	1.11 (0.69–1.79)	1.15 (0.71–1.86)	1.23 (0.66–2.30)	1.33 (0.69–2.56)	1.47 (0.75–2.90)
Upper college	0.70 (0.41–1.22)	0.80 (0.44–1.45)	0.87 (0.47–1.60)	1.05 (0.51–2.13)	1.53 (0.72–3.25)	1.82 (0.84–3.94)

Model 1: Education

Model 2: Education and BMI

Model 3: Education, BMI, marital status and having children

*OR *odds ratio, *CI 95%* 95% confidence interval

**p < 0.01; *p < 0.05

^a^Interaction between gender and education *p* < 0.05

In women, the proportion of those having reported body dissatisfaction increased from 14.2 to 22.4% between the ages of 22 and 52, and in men, the corresponding increase was from 7.5 to 11.9%. The effect of time on the increase in body dissatisfaction was significant in both women (OR = 1.23, *p* < 0.001) and men (OR = 1.19, *p* < 0.01). When BMI was added to the model, the effect of time turned to the opposite direction in women (OR = 0.83, *p* = 0.001), whereas in men, the effect turned non-significant (OR = 0.91, *p* = 0.213).

**Fig. 1:**
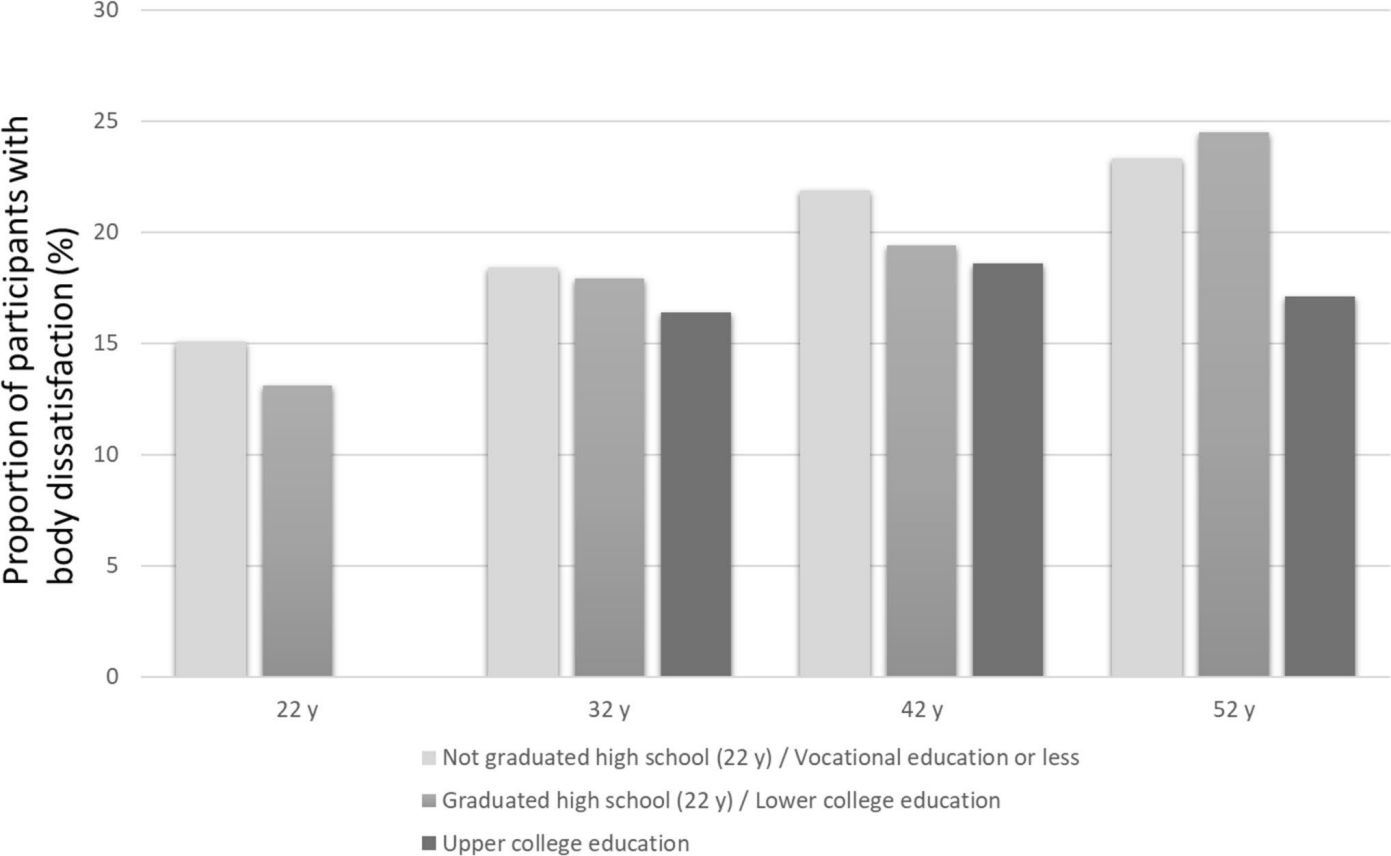
Body dissatisfaction at ages 22, 32, 42, and 52 by education in women

**Fig. 2:**
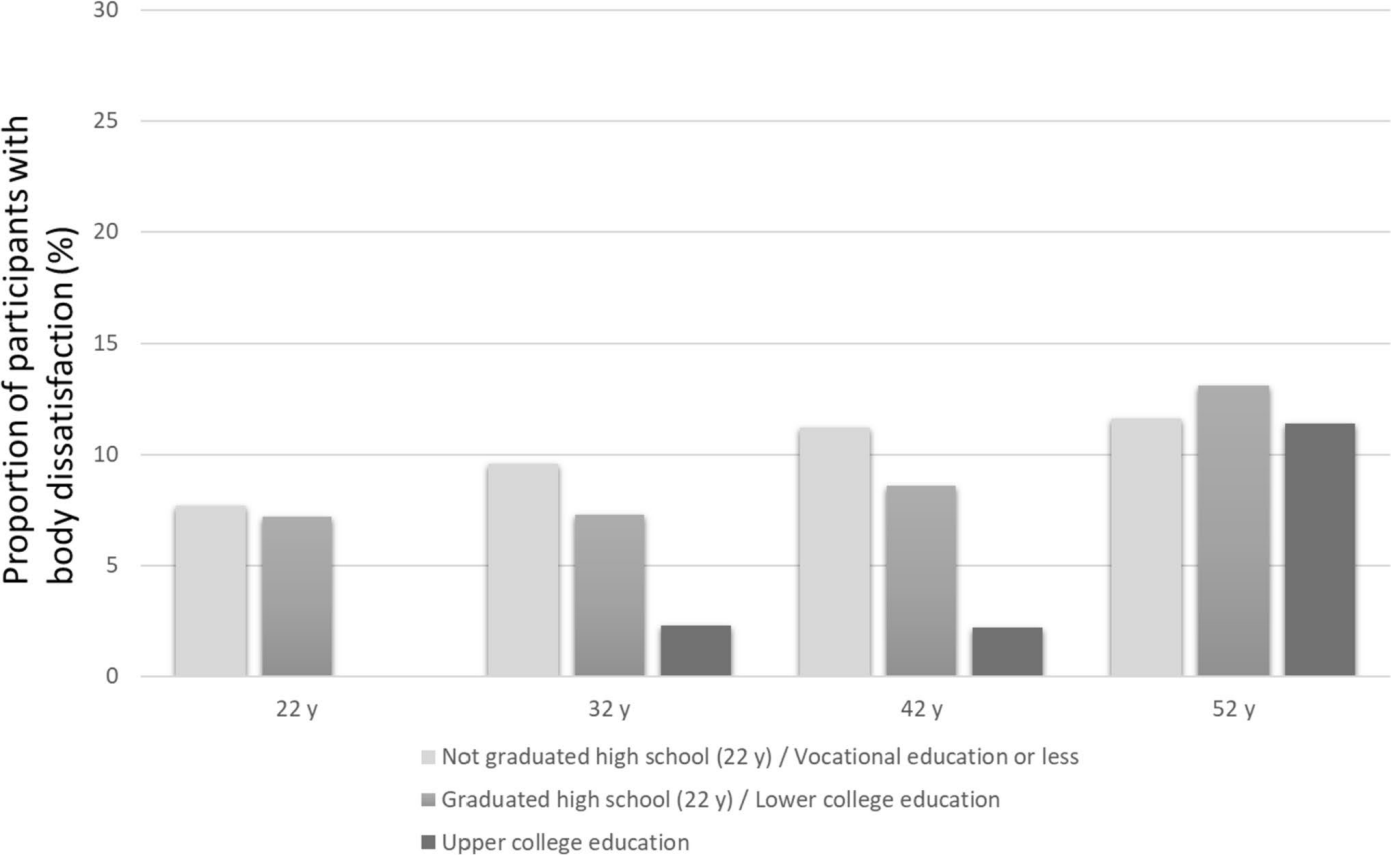
Body dissatisfaction at ages 22, 32, 42, and 52 by education in men

Figures 1 and 2 show the proportions of women and men with body dissatisfaction in different educational groups at different ages. The figures illustrate that the level of body dissatisfaction appeared lower in higher educational groups. The difference was especially large among 32- and 42-year-old men, among whom those with upper college education had a remarkably lower amount of body dissatisfaction compared to the men in the two lower educational groups (Fig. 2). By the age of 52, the SES differences in men’s body dissatisfaction seemed to have disappeared. The differences in the effects of education (lowest vs. highest) on body dissatisfaction between timepoints were also significant among men (*p* < 0.05).

The associations between body dissatisfaction and educational level at different ages were further explored using logistic regression (Table 2). In women, there were no significant associations between body dissatisfaction and educational level at any age. Among men at the ages of 32 and 42, those with upper college education had lower odds of reporting body dissatisfaction compared to those with vocational education or less (model 1). The associations remained significant when the model was controlled for BMI (model 2) as well as for BMI, marital status, and having children (Model 3), indicating that the associations were independent of these variables. The interaction terms between gender and upper college education were significant at the ages of 32 and 42, indicating that at those ages, the association of education with body dissatisfaction was different between women and men.

**Table 3:** Body dissatisfaction during the life course by educational attainment by age 52 among women and men. Multinomial logistic regression

	**Women**			**Men**		
	**Model 1** **(*N* = 881)**	**Model 2** **(*N* = 867)**	**Model 3** **(*N* = 756)**	**Model 1** **(*N* = 738)**	**Model 2** **(*N* = 735)**	**Model 3** **(*N* = 611)**
	**RRR (CI 95%)**	**RRR (CI 95%)**	**RRR (CI 95%)**	**RRR (CI 95%)**	**RRR (CI 95%)**	**RRR (CI 95%)**
**Steady positive body image (reference category)**						
**Steady neutral body image**						
Vocational education or less 22–52 y						
At least lower college education completed 22–52 y	0.69 (0.49–0.95)*	0.72 (0.51–1.01)	0.73 (0.51–1.06)	0.61 (0.45–0.83)**	0.62 (0.45–0.84)**	0.55 (0.39–0.78)**
**Increasing body dissatisfaction**						
Vocational education or less 22–52 y						
At least lower college education completed 22–52 y	0.67 (0.46–0.99)*	0.72 (0.48–1.10)	0.72 (0.45–1.15)	0.63 (0.36–1.11)	0.65 (0.36–1.16)	0.51 (0.25–1.02)

Model 1: Lower college education or higher completed 22–52 years

Model 2: Lower college education or higher completed 22–52 years and mean BMI 22–52 years

Model 3: Lower college education or higher completed 22–52 years, mean BMI 22–52 years, being married or cohabiting in most of the follow-ups 22–52 years and having children in any of the follow-ups 22–52 years

No significant interactions (*p* < 0.05) between gender and education were found

*RRR* relative risk ratio, *CI 95%*, 95% confidence interval

**p < 0.01; *p < 0.05

**Fig. 3:**
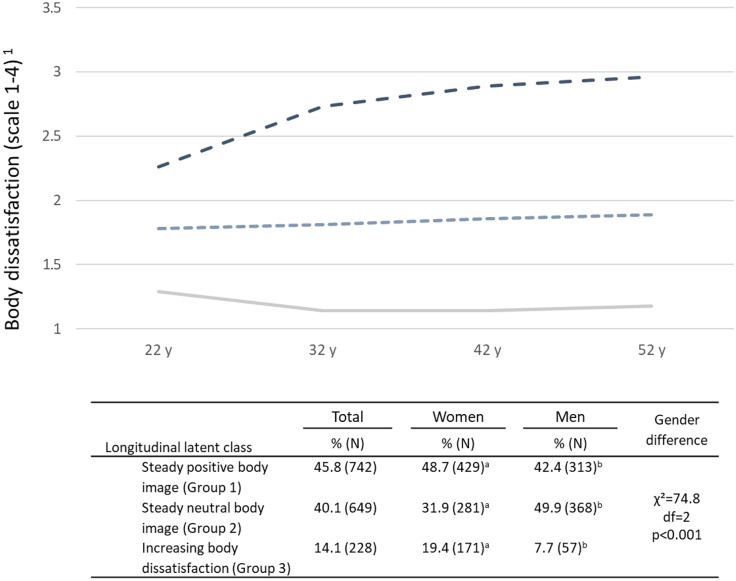
Three class solutions from longitudinal latent class analyses of body satisfaction. Different superscript letters (a, b) denote significant (*p* < 0.05) differences in the proportions of a given latent class between men and women. ^1^A four-category variable of body dissatisfaction: 1, I am fairly satisfied with my appearance; 2, There are no physical traits that bother me in my appearance; 3, I am worried that I look unattractive; 4, I feel that I look ugly/I am sure that I look ugly and disgustin

In the next step, latent class analyses were run to identify longitudinal latent classes of body dissatisfaction. The three-group solution was considered to best fit the data based on the statistical criteria (see Appendix Table 4) and also to be most informative. Figure 3 visualizes the latent trajectory groups representing the development of body (dis)satisfaction over the life course. Group 1 represents a steady positive body image over the life course, with the respondent having typically reported body dissatisfaction at level 1 (*I am fairly satisfied with my appearance*). Group 2 represents a steady neutral body image, with typically reporting body dissatisfaction at level 2 (*There are no physical traits that bother me in my appearance*)*.* In those who were most likely in group 3, body dissatisfaction increased over the life course, so that the proportions of reporting body dissatisfaction at level 3 (*I am worried that I look unattractive*) and at level 4 (*I feel that I look ugly/I am sure that I look ugly and disgusting*) increased with age. Women most typically belonged into the group of steady positive body image and men into the group of steady neutral body image, while belonging to the increasing body dissatisfaction group was more common in women compared to men. Gender differences in trajectory group proportions were statistically significant (Fig. 3).

The latent body (dis)satisfaction groups were predicted with educational attainment (whether the respondent had completed at least lower college education during the follow-up or not) using multinomial regression models (Table 3). Among women, those who had completed at least lower college education during the follow-up had lower risk for belonging to the groups of both steady neutral body image and increasing body dissatisfaction, compared to the group of steady positive body image (model 1). However, when adjusting the models with mean BMI during follow-up (model 2), the associations attenuated and turned non-significant.

In men, those who had completed at least lower college education during the follow-up had lower risk for belonging to the group of steady neutral body image than into the group of steady positive body image, compared to those men who had not achieved at least lower college education. This association remained significant when adjusting the model with mean BMI (model 2) and with aggregate variables of marital status and having children (model 3), indicating that the association was independent from those variables. For the group of increasing body dissatisfaction, the relative risk ratios (RRR) were quite similar to those for the group of steady neutral body image, but not statistically significant (models 1–3). There were no significant gender differences in the association between body dissatisfaction trajectory classes and education (models 1–3).

## Discussion

Our study examined the associations between body dissatisfaction and SES, measured as educational attainment, over the life course in a population-based cohort of Finnish women and men. Contrary to our hypothesis, the results suggested that body dissatisfaction is associated with lower education if anything. Body dissatisfaction was associated with lower education in men at the ages of 32 and 42, and furthermore, men with lower education were more likely to maintain a less positive body image over the life course. Among women, having completed a lower educational level during the follow-up did predict the development of higher body dissatisfaction over the life course, but the associations were no more significant when BMI was taken into account.

The overall prevalence of body dissatisfaction ranged from 14 to 22% among women and from 8 to 12% among men, indicating that the majority of participants did not report body dissatisfaction. In a review by Fiske et al. [34], the prevalence of body dissatisfaction was found to vary widely, between 11–72% in women and 8–61% in men, with the prevalence observed in our study being at the lower end of this range. Body dissatisfaction was more common among women than men throughout the life course regardless of educational background. The fact that women are more dissatisfied with their bodies than men has been widely acknowledged in previous studies as well [11, 14, 15]. This has mainly been explained by society’s stricter appearance norms for women [1] and the pressure for women to maintain a youthful appearance as they age [35]. We found that women’s body dissatisfaction increased with age. However, when BMI was added to the model, the direction of the association was reversed, and body dissatisfaction appeared to decline with age. This is an interesting finding and somewhat contradictory to previous findings showing a relatively stable amount of body dissatisfaction among women during adulthood, turning into decline not until older age, when body image becomes a less important aspect in women’s life. Our finding, nevertheless, becomes understandable in the light of the facts that the current body ideal for women is very slender [7] and further emphasizes the central role weight has in Western culture’s beauty models for women. Moreover, our study provided indications that women in all SES groups would be equally vulnerable to body dissatisfaction through the follow-up. Women with lower education did develop a slightly poorer body image during the follow-up, but there was no significant difference between different educational groups when BMI was added to the model.

Body dissatisfaction increased with age also among men, although not as strongly as among women. In previous studies, men’s body dissatisfaction has generally not been found to clearly increase or decrease with age, but rather to change form during adulthood, with young men desiring to be more muscular and older men wanting to lose weight [11, 15, 19]. Among men, body dissatisfaction was more common in lower educational groups at ages of 32 and 42. The effects of SES on body image appeared to be far-reaching: men who had not completed at least lower college education during the follow-up were more likely to maintain a steady neutral than steady positive body image during the follow-up. A similar difference was also found between those with increased body dissatisfaction and those who were maintaining a steady positive body image over the life course, but the results were not statistically significant, probably due to reduced statistical power. Our findings were contradictory to our hypothesis, which suggested that body dissatisfaction would have been more common in higher SES groups, as found in some of the previous studies [8, 25, 26]. Interestingly, in cross-sectional examinations, the SES differences in men’s body dissatisfaction disappeared at the age of 52, as there was an increase in body dissatisfaction among men in the upper college education group. The strong increase in highly educated men’s body dissatisfaction during middle age raises the question of whether highly educated men would be under higher pressure to maintain a youthful and dynamic appearance as they age. When examining men’s body image, it is also relevant to note that men have been assessed to underreport their body dissatisfaction which is generally held as a feminine phenomenon [36, 37].

Although the findings associating body dissatisfaction with lower education in men were inconsistent with our hypothesis, it is worth noting that the research on the subject has been rare and has provided rather conflicting results also in the past. A couple of previous studies have similarly associated body dissatisfaction to lower SES, although these studies have been conducted among adolescents and young adults [28, 29]. It has also been pointed out in the contemporary body image literature that due to the enormous cultural impact of mass media, one of the main distributors of the unrealistic Western beauty ideals, the appearance norms and body image problems have extended to every stratum of society instead of being only an upper-class phenomenon [1, 7], an assumption that has been prominent in traditional sociological theories [e.g., 27]. Our participants were living their young adulthood in the 1980s and reached middle age during the current millennium, during which some major societal changes in appearance ideals have taken place, especially with regard to increased appearance pressures for men [2, 7]. Therefore, a long cohort study is a particularly interesting contribution to the study field, enabling us to investigate the development of body dissatisfaction in a design that incorporates both the changes in the body caused by aging and the societal changes that are meanwhile taking place in the environment.

As mentioned, the longitudinal 30-year follow-up was a key strength of our study, allowing us to study the development of body dissatisfaction with age within the same cohort. Another notable strength was the inclusion of men, as well as middle-aged men and women, as body image research has traditionally focused on young women. Furthermore, using the same measure for body dissatisfaction in all the measurement points enabled comparability of the results between time points. However, there were also some limitations in our study that need to be considered. One central limitation of this study is the rather simplistic measure of body dissatisfaction, which was based on a single-item evaluation of one’s satisfaction with his/her appearance and was not capturing the various aspects of body image, such as the importance of muscularity in men’s body dissatisfaction [15]. Nevertheless, appearance satisfaction is one of the various ways in which body image has been conceptualized in the research literature [7], and some of these studies have also used single-item measures [e.g., 38, 39]. When making comparisons between genders, it is also worth noting that the item in question measuring body dissatisfaction in Beck Depression Inventory may work in different ways for women and men [40]. Also, as the results were obtained by measuring SES through self-reported education, they do not necessarily generalize to results obtained by the other commonly used SES measures (income level and occupation), as they have been found to be partially separate constructs [21]. Furthermore, in some of the analyses, the subgroups were rather small, leading to a decrease in statistical power. Finally, in terms of generalizability of the results, it should be noted that the sample was from a Nordic welfare country.

In conclusion, our prospective longitudinal study design with 30 years of follow-up provides a novel developmental insight to the study of body image and SES. Body dissatisfaction increased with age in both women and men, although this seemed to be explained by an increase in BMI. In women, the association even reversed, highlighting the importance of BMI in women’s body image — otherwise, women might be quite satisfied with their appearance as they age. Furthermore, our results suggest body image problems to be more common in lower educational groups during adulthood in men. The development of body dissatisfaction was associated with lower education also among women, but only until considering the higher BMI of lower educational groups. Given that a positive body image is associated with better physical and mental health, higher self-esteem, and healthier eating habits [41], the differences between educational groups in body dissatisfaction are important to consider when planning health interventions to different SES groups. To this end, our results highlight the importance of cultural, social, and gender sensitivity in reducing body dissatisfaction.

## Data Availability

The data underlying this article cannot be shared publicly due to legal restrictions (Finnish Data Protection Act 1050/2018) and the nature of the data (individual level data). Data are available upon request. Data requests are reviewed in Finnish Institute for Health and Welfare for compliance with the original research purposes of the study project. Requests are subject to legal restrictions of the Finnish Data Protection Act.

## Appendix

**Table 4:** Statistics for longitudinal latent class solutions by requested number of latent classes

No of classes	Log-likelihood	AIC	BIC	A-BIC	VLMR p	LMR-A p	BLRT p	Entropy	No of cases in classes
1	− 5550.4	11,124.8	11,189.5	11,151.4					1619
2	− 5224.4	10,498.7	10,633.5	10,554.1	0.0000	0.0000	0.0000	0.596	937, 682
3	− 5086.4	10,248.7	**10,453.5**	10,332.8	**0.0000**	**0.0000**	0.0000	0.627	742, 649, 228
4	− 5057.9	**10,217.9**	10,492.7	**10,330.7**	0.9870	0.9884	**0.0000**	0.676	742, 624, 213, 40
5^a^	− **5048.7**	10,225.5	10,570.4	10,367.1	1.0000	1.0000	1.0000	0.632	603, 572, 222, 180, 42

*AIC* Akaike information criterion, *BIC* Bayesian information criteria, *A-BIC* sample-size adjusted BIC, *VLMR* Vuong-Lo-Mendell-Rubin likelihood ratio test (n-1 (H0) vs. n classes), *LMR-A* Lo-Mendell-Rubin adjusted likelihood ratio test, *BLRT* bootstrapped likelihood ratio test

^a^Problems in model estimation
